# Copper and nanostructured anatase rutile and carbon coatings induce adaptive antibiotic resistance

**DOI:** 10.1186/s13568-022-01457-z

**Published:** 2022-09-07

**Authors:** Alibe Wasa, Jack Aitken, Hyunwoo Jun, Catherine Bishop, Susan Krumdieck, William Godsoe, Jack A. Heinemann

**Affiliations:** 1 School of Biological Sciences, University of Canterbury, Canterbury, New Zealand; 2 Department of Mechanical Engineering, University of Canterbury, Canterbury, New Zealand; 3 Bio-Protection Centre, Lincoln University, Canterbury, New Zealand; 4 Present Address: School of Energy, Geoscience, Infrastructure and Society, Heriot Watt University, EH14 4AS Edinburgh, UK

**Keywords:** Antimicrobial coatings, Copper, TiO_2_, Antibiotic resistance

## Abstract

Contaminated surfaces are vehicles for the spread of infectious disease-causing microorganisms. A strategy to prevent their spread is applying antimicrobial coatings to surfaces. Both nanostructured anatase rutile and carbon (NsARC), a TiO_2_ formulation, and copper are examples of antimicrobial agents that are used in making or coating door handles and similar surfaces, to reduce microbial loads. Antimicrobial surfaces have been extensively tested for antimicrobial activity but not sublethal effects, such as exposure-associated multiple antibiotic resistance phenotypes usually caused by induction of efflux pump genes. The possibility of NsARC and copper inducing indicative efflux pump pathways was investigated by monitoring the expression of mScarlet fluorescent protein (FP) in two reporter strains of *Escherichia coli.* There was an increase in the expression of FP in the reporter strains exposed to NsARC and copper relative to the inert control composed of stainless steel. Furthermore we tested *E. coli* and *Staphylococcus aureus* following 8 h of exposure to NsARC for changes in resistance to selected antibiotics. *E. coli* that were exposed to NsARC became more susceptible to kanamycin but there was no significant change in susceptibility of *S*. *aureus* to any tested antibiotics. These findings suggests that even though NsARC and copper are antimicrobial, they also have some potential to cause unintended phenotypes.

**Supplementary Information:**

The online version contains supplementary material available at 10.1186/s13568-022-01457-z.

## Keypoints

1. New antimicrobial material NsARC based on TiO_2_ compared to copper.
2. Both NsARC and copper induce efflux pump gene transcription.
3. Species-specific changes in antibiotic resistance from exposure to antimicrobial surface coatings.

**Supplementary Information:**

The online version contains supplementary material available at 10.1186/s13568-022-01457-z.

## Introduction

Nanostructured anatase rutile and carbon (NsARC), a composite of titania and carbon is an antimicrobial surface coating that is active with or without photoactivation (Wasa et al. 2021). The killing mechanism of NsARC has not been precisely described but at least involves free radicals that are released sequel to photoexcitation of TiO_2_ (Krumdieck et al. 2019), active carbon-centred free radicals (Fenoglio et al. 2009) and desiccation of cells over time (Yu et al. 2014). NsARC with antimicrobial properties could be of value because disease-causing microorganisms are becoming resistant to other available methods of prevention and treatment (Djurišić et al. 2015; Leung et al. 2016). Prescription medicines, disinfectants, and other antimicrobial agents are not uniformly effective against disease-causing microorganisms and the use of any can select for reduced susceptibility to the others (Dancer 2008; Foster et al. 2011; Kampf 2018). Therefore, the diseases caused by these microorganisms are becoming more difficult, sometimes impossible, to treat with antibiotics (Heinemann 1999; Metcalfe et al. 2016; Williamson et al. 2015).

Exposure of microorganisms to toxic metals is unavoidable and desirable. Toxic metals are sometimes found in high concentrations in the environment as a result of natural geological events such as volcanos and or human activities such as mining, smelting, fossil fuel burning and other industrial activities (Ali et al. 2019; Barkay et al. 2010). Microorganisms have evolved mechanisms by which they can both acquire and maintain essential metals at physiologically relevant concentrations, and eliminate them when they are in excess (Ali et al. 2019; Chandrangsu et al. 2017). They have a variety of strategies for limiting toxicity such as extracellular and or intracellular sequestration of metals, modification of target sites, reduction in outer membrane porins (OMP), enzymatic detoxification and or increase in efflux of metals (Nies 2003), and biofilm formation. Resistance to nanomaterials such as NsARC might be more difficult for microorganisms to evolve. Nanomaterials can have multiple cellular targets and therefore the options available for microorganisms to mitigate the effects of nanomaterials is limited (Lemire et al. 2013; Wright et al. 2006).

Nevertheless, sub-lethal exposures might still result in unintended effects as has been observed for other antimicrobial agents. Exposing bacteria to non-antibiotic chemicals can predispose them to develop resistance to antibiotics (Jun et al. 2019; Kurenbach et al. 2015, 2017, 2018). *Salmonella enterica* serovar Typhimurium exposed to sublethal concentration of the herbicides Kamba^®^ and 2,4-D grew at higher concentrations of ampicillin, chloramphenicol, ciprofloxacin and tetracycline antibiotics (Kurenbach et al. 2015). *Escherichia coli* exposed to the fungicide copper ammonium acetate also grew on medium with higher concentrations of tetracycline and *E. coli* exposed to atrazine grew on higher concentrations of ciprofloxacin, kanamycin and streptomycin (Jun et al. 2019). Food preservatives and emulsifiers used in food and medicines could also cause these types of changes (Kurenbach et al. 2017; Molina-González et al. 2014). The way bacteria respond after exposure to various chemicals may vary depending on their genetic and physiological differences (Chiang and Schellhorn 2012).

Should NsARC be used on a commercial scale for its antimicrobial properties, we wanted to know if there were unintended outcomes that we could anticipate. We previously hypothesized that sub-lethal exposures to NsARC could cause similar changes to antibiotic susceptibility (Wasa et al. 2021). This is based on the innate response of bacteria to toxic environments. The innate response includes but is not limited to changes in expression of efflux and permeability. Common markers for this response are *tolC* and *soxS* genes (Alderton et al. 2021). Changes in the expression of these two genes can alter susceptibility pattern of *E. coli* to antibiotics. This was investigated comparing NsARC, copper and stainless steel.

## Materials and methods

### Bacterial strains, culture conditions, materials and chemicals

Bacterial strains used in this study are shown in Table 1. *E. coli* BW25113 (Baba et al. 2006) is a useful strain for genetic studies because it the parent in a series that includes nearly every possible gene knockout. We have measured the baseline responses of efflux phenotypes and gene expression using this strain (Kurenbach et al. 2017). Strains are stored in 15% (v/v) glycerol solution at − 80 °C. They were recovered for use on Lauria Bertani (LB) agar plates (Lennox-L-Broth Base, Invitrogen, Auckland (New Zealand) and agar (Bacteriological Agar No.1, Oxoid, Hampshire (UK)) and then incubated at 37 °C. Plates were replaced at one-week intervals. All antibiotics were purchased from Sigma, Auckland (New Zealand).

**Table 1:** Bacterial strains used in this study

	Characteristics/genotype	References
*Staphylococcus aureus* ATCC25923	agr-III strain	Mun et al. (2013)
*Escherichia coli* BW25113, CGSC#: 7636	F-, λ-, Δ(*araD-araB*)567, Δ*lacZ*4787*(::rrnB-*3), *rph-*1,Δ(*rhaD-rhaB*)568, *hsdR*514	Baba et al (2006)
Plasmids		
pFru-p_tolC_-mScarlet	Kan^R^, cat, nptII, pBBR1, *tolC* promoter:mScarlet-IE	Jun et al. (2019)
pFru-p_SoxS_-mScarlet	Kan^R^, cat, *nptII, pBBR*1, *soxS* promoter:mScarlet-I	Jun et al. (2019)
*Escherichia coli* with plasmids		
BW*tolC*	BW25113 (pFru-p_tolC_-mScarlet)	Jun et al. (2019)
BW*soxS*	BW25113 (pFru-p_soxS_-mScarlet)	Jun et al. (2019)

Paraformaldehyde used for fixation of cells prior to fluorescence microscopy was purchased from Sigma, Auckland (New Zealand) and stored as powdered stock at 4 °C. 4% (v/v) solution of paraformaldehyde was made up on the day it was to be used and kept at 4 °C.

Stainless steel (25 × 25 mm) coupons were used as an inert control material as recommended by the ISO 27447:2009 (Mills et al. 2012), and commercially pure copper (25 × 25 mm) coupons were selected as a positive control material because they have known antimicrobial properties that are independent of light (Wasa et al. 2021). The NsARC (25 × 25 mm) coupons and control (stainless steel and copper) samples were sterilised by immersion in 70% ethanol and the NsARC test pieces were then aseptically stored in the dark for > 48 h. Gelatine coated slides were made by immersing glass microscopy slides (Mareinfeld-Superior, 76 × 26 mm, approx. 1 mm thick) in 70% ethanol for 1 h, then air drying and dipped in 0.1% (v/v) gelatine solution at 70^o^C before air drying again. After drying the slides were kept at 4 °C and used within 1 week of drying.

### Determining if NsARC can cause a change in susceptibility of ***E***. ***coli*** and ***S***. ***aureus*** to antibiotics

The *E. coli* culture was exposed to the tests and control metal samples using a modified version of ISO 27447:2009 (Wasa et al. 2021). 100 µl of the test organisms containing ~ 1,000,000 cells at the stationary growth phase were placed on 25 × 25 mm NsARC and stainless-steel surfaces and 24 × 24 mm sterile coverslip (Lab Supply, Dunedin (New Zealand)) was place on top, causing the liquid to spread evenly on the surface. The samples were then placed in 60 × 15 mm petri dishes. Replicates were then simultaneously exposed to visible light of 2100 lx (450–650 nm) or kept in the dark for 8 h. Afterwards, the samples were rinsed using phosphate buffered saline (PBS). The number of bacteria in 100 µl of the PBS was about ~ 10^2^ cells. This volume was transferred to the surface of LB plates containing 0, 0.01, 0.02, 0.03, 0.04, 0.05 µg/ml of ciprofloxacin, or 0, 5, 6, 7, 8, 9 µg/ml of chloramphenicol, or 0, 4, 5, 6, 8, 9 µg/ml of kanamycin or 0, 0.5, 0.7, 1, 1.5, 2 µg/ml of tetracycline.

The same procedure as followed using *Staphylococcus aureus*. Wash off from NsARC was transferred to the surface of LB plates containing 0, 1, 2, 3, 4, 5, 6 µg/ml of erythromycin, or 0, 0.09, 0.1, 0.2, 0.3, 0.4 µg/ml of fusidic acid, or 0, 0.9, 1, 2, 3, 4 µg/ml of kanamycin, or 0, 0.07, 0.08, 0.09, 0.1, 0.2 µg/ml of oxacillin, or 0, 0.09, 0.1, 0.2, 0.3, 0.4 µg/ml of tetracycline or 0, 0.5, 0.8, 1, 1.5, 2 µg/ml of vancomycin. The *E. coli* and *S. aureus* wash off from stainless steel was diluted threefold to achieve ~ 10^2^ cells and then was transferred to the surface of LB plates containing various concentration of antibiotics as described above. The minimum inhibitory concentrations of the antibiotics are shown in Additional file 1: Table S21. Plates were incubated at 37 °C for 24 h. All experiments were conducted three times to obtain biological replicates. Three samples of each (test and control) were used for each experiment to obtain technical replicates. The plates were monitored for up to three days. This was compared for the various treatments (material and exposure conditions). There were variations in the values, thus, the cfu/ml counts were normalised to efficiency of plating (EOP) values using the formula.

$${\rm EOP} =\frac{titre\,of\,treatments\,(\text{L}\text{B} + \text{a}\text{n}\text{t}\text{i}\text{b}\text{i}\text{o}\text{t}\text{i}\text{c}\text{s})}{titre\,of\,control \left(LB\,only \right)}.$$

The EOP values were then used to plot graphs using graphpad prism software.

### Determining if NsARC can induce gene expression

Individual colonies formed by reporter strains *E*. *coli BWtolC* and *BWsoxS* (Jun et al. 2019) were placed into LB broth (Lennox-L-Broth Base, Invitrogen, Auckland (New Zealand)) supplemented with kanamycin and then placed on a shaker platform at 37 °C and grown to exponential phase. 100 µl containing about ~ 10^7^cells was then placed on separate NsARC (test), stainless steel (negative control) and copper (positive control) coupons. Sterile cover slips (24 mm × 24 mm) were used to spread the cultures on the sample surfaces. The samples were placed in petri dishes (60 mm × 15 mm) containing damp filter paper. Replicates were simultaneously exposed to visible light of 2100 lx (450–650 nm), UV light (365 nm), ambient light (650–750 nm) and also kept in the dark for a period of not more than 2 h before washing off with PBS and the cells fixed with paraformaldehyde (Chao and Zhang 2011). 2 µl of the fixed cells were then smeared onto a gelatine-coated glass slide and allowed to dry at room temperature. 6 µl of 50% (v/v) glycerol was then added to the dried smear and a 25 × 25 mm glass cover slip was used to spread it over the dried smear.

### Microscopy and image processing

The fixed cells were examined with an Axio Imager.M1 (Zeiss, Oberkochen, Germany) using 556/20 nm excitation bandpass. Digital images were captured at 100⋅ magnification with an AxioCam MRm camera (Zeiss) in phase contrast and through a 556/20 nm (red) filter set. Approximately 10 NsARC and 10 control samples each were used for this experiment. And about 50 images were captured from each individual sample. A total of 500 images from each treatment was taken for each treatment replicate. Images were then analysed using Fiji ImageJ (Schindelin et al. 2012). 100 separate images for each sample were analysed. Single-cell fluorescence intensity of the individual cells was obtained by acquiring multichannel images of the fluorescence signals and phase contrast signals. Multichannel images were imported into the Fiji ImageJ program. Thresholding command and standard settings were used to separate cells from the background based on the phase contrast. The analyse particles command was then used to add cells to the region of interest manager and the average fluorescence of the individual cells was determined using the “multi-measure” command (Remus-Emsermann et al. 2016). The relative fluorescence is also just an arbitrary unit (au) of measurement.

### Statistical analysis

R was used for statistical analysis (Rosario-Martinez et al. 2015). We used analysis of variance (ANOVA) to analyse the data from the experiment we carried out to determine if exposure of bacteria to NsARC caused a change in susceptibility to antibiotics. A multifactor ANOVA was performed on EOP values to test for effect of the materials (NsARC and control) and antibiotic concentration under two exposure conditions (light and dark). Residual plots were examined to determine if EOP values were normally distributed, which is an assumption for ANOVA (Crawley 2007). The plots were not normally distributed. So, the EOP scores were log transformed to meet the assumption. In each case, we tested for significant difference between materials. The null hypothesis was that there was no difference between the EOP values from the materials at various antibiotic concentrations. We also tested for interaction between materials, antibiotic concentrations and exposure conditions. A Bonferroni’s post hoc test was used to compare the EOP to determine if there is a significant difference between NsARC and the controls. The value for statistical significance was set at *P* < 0.05. The results of each post hoc are available in supplementary material (Additional file 1: Table S1–S12), however, we were most interested in the differences in EOP between individual treatment combinations as follows: NsARC vs. steel under light and NsARC vs. steel in the dark. Contrast matrices listing the contrast of interest mentioned were drawn up and the test Interactions function in the phia package in R was used to evaluate the contrasts as described in the result section (Rosario-Martinez et al. 2015).

For the experiment to determine if NsARC can induce changes in the expression pattern of genes that can alter susceptibility to antibiotics, a statistical model based on ANOVA was also used. A Tukey’s post hoc test was used to compare the means of the relative fluorescence to determine if there is a difference between NsARC and the controls. The value for statistical significance was set at *P* < 0.05. The results of each post hoc are available in supplementary material (Additional file 1: Table S13–S20). However, we were most interested in the differences between individual treatment combinations, these include NsARC vs. Positive control (copper), Negative control (stainless steel) vs. Positive control (copper) and NsARC vs. Negative control (stainless steel). These were calculated using contrasts and the results can also be found in the supplementary materials (Additional file 1: Table S13–S20). Violin plots were then made using ggplot2 (Wickham 2016).

## Results

### Change in susceptibility to antibiotics

**Fig. 1:**
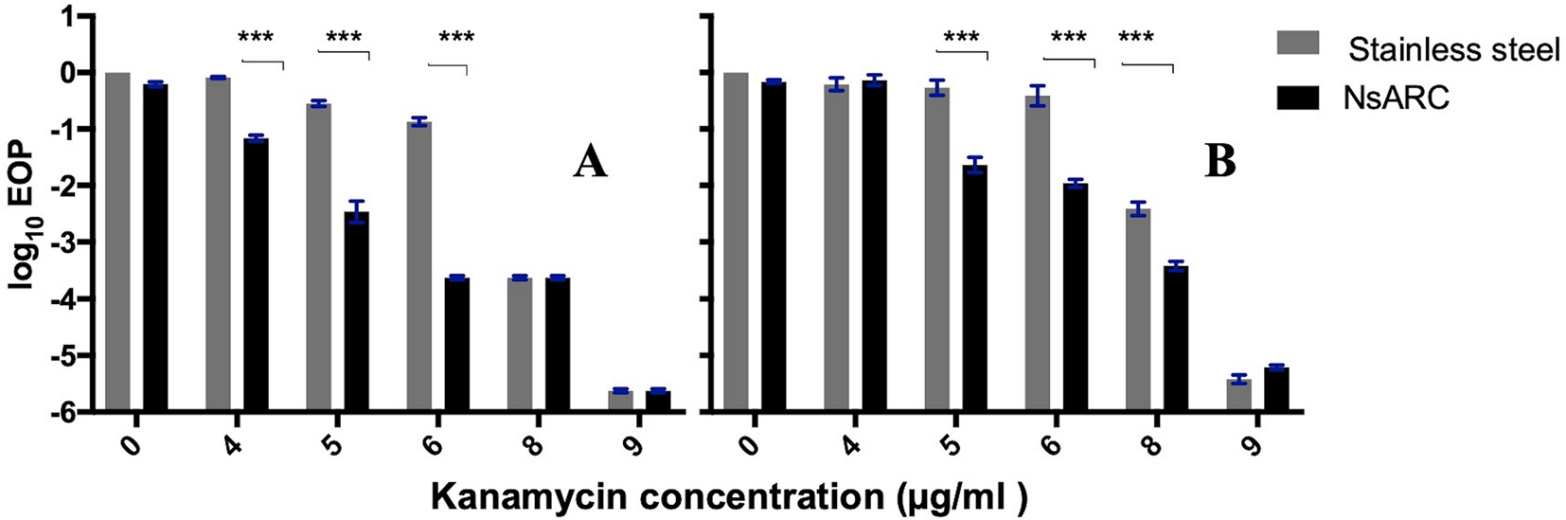
Response of *E. coli* to various concentrations (µg/ml) of kanamycin after exposure to stainless steel and NsARC (**A**) under visible light (**B**) in the dark. Error bars are standard error of means (SEM). Asterisks indicate P values. *P < 0.05; **P < 0.01; ***P < 0.001; *NS* not significant

**Fig. 2:**
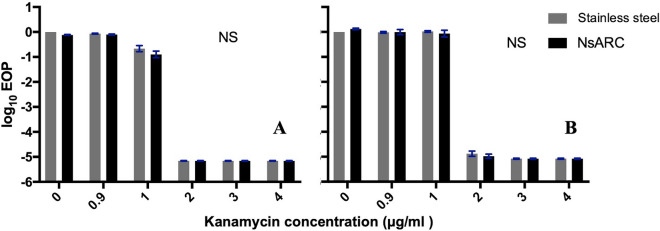
Response of *S. aureus* to various concentrations (µg/ml) of kanamycin after exposure to stainless steel and NsARC (**A**) under visible light (**B**) in the dark. Error bars are standard error of means (SEM). Asterisks indicate P values. *P < 0.05; **P < 0.01; ***P < 0.001; *NS* not significant

10^2^ cells of *E. coli* and *S. aureus* that had survived exposure to NsARC with and without light for about 8 h were tested for responses to selected antibiotics. *E. coli* were challenged with different concentrations of ciprofloxacin, chloramphenicol, kanamycin, and tetracycline and *S. aureus* with different concentrations of kanamycin, vancomycin, erythromycin, oxacillin, tetracycline and fusidic acid. Changes in response to each antibiotic because of the previous exposure to NsARC were shown as a differential efficiency of plating (EOP) (Figs. 1, 2). No significant change in susceptibility to tetracycline (Additional file 1: Table S3), chloramphenicol (Additional file 1: Table S4) and ciprofloxacin (Additional file 1: Table S5) was observed (Additional file 1: Fig. S1). A significant change (*P* < 0.001) in the EOP was observed for *E. coli* exposed to both light and NsARC or only NsARC (Fig. 1). The significant change was in the direction of lowering the minimum inhibitory concentration of kanamycin after exposure to NsARC.

No significant change in susceptibility to any antibiotic was observed for *S. aureus* that had survived exposure to NsARC with or without light (Fig. 2; Additional file 1: Fig. S2).

### *tol*C and *sox*S can be induced by NsARC

Two reporter strains each expressing either *PtolC-*mScarlet or *PsoxS-*mScarlet were designed and constructed as described previously (Jun et al. 2019). These two strains of *E. coli* “report” changes in transcription of the *tolC* and *soxS* genes. We and others have found that these genes are often induced in response to stress (Fernández and Hancock 2012), particularly oxidative stress (Demple 1996).

Each of these reporter strains had a plasmid construct that expressed an mScarlet red fluorescent protein under the control of the promoters of either the *tolC* or *soxS* genes. These designed constructs are strains wherein an increase in expression of fluorescent protein is expected when the *tolC* or *soxS* genes are induced (Jun et al. 2019). After exposure to NsARC, stainless steel and copper, the single cell fluorescence intensities were measured.

**Fig. 3:**
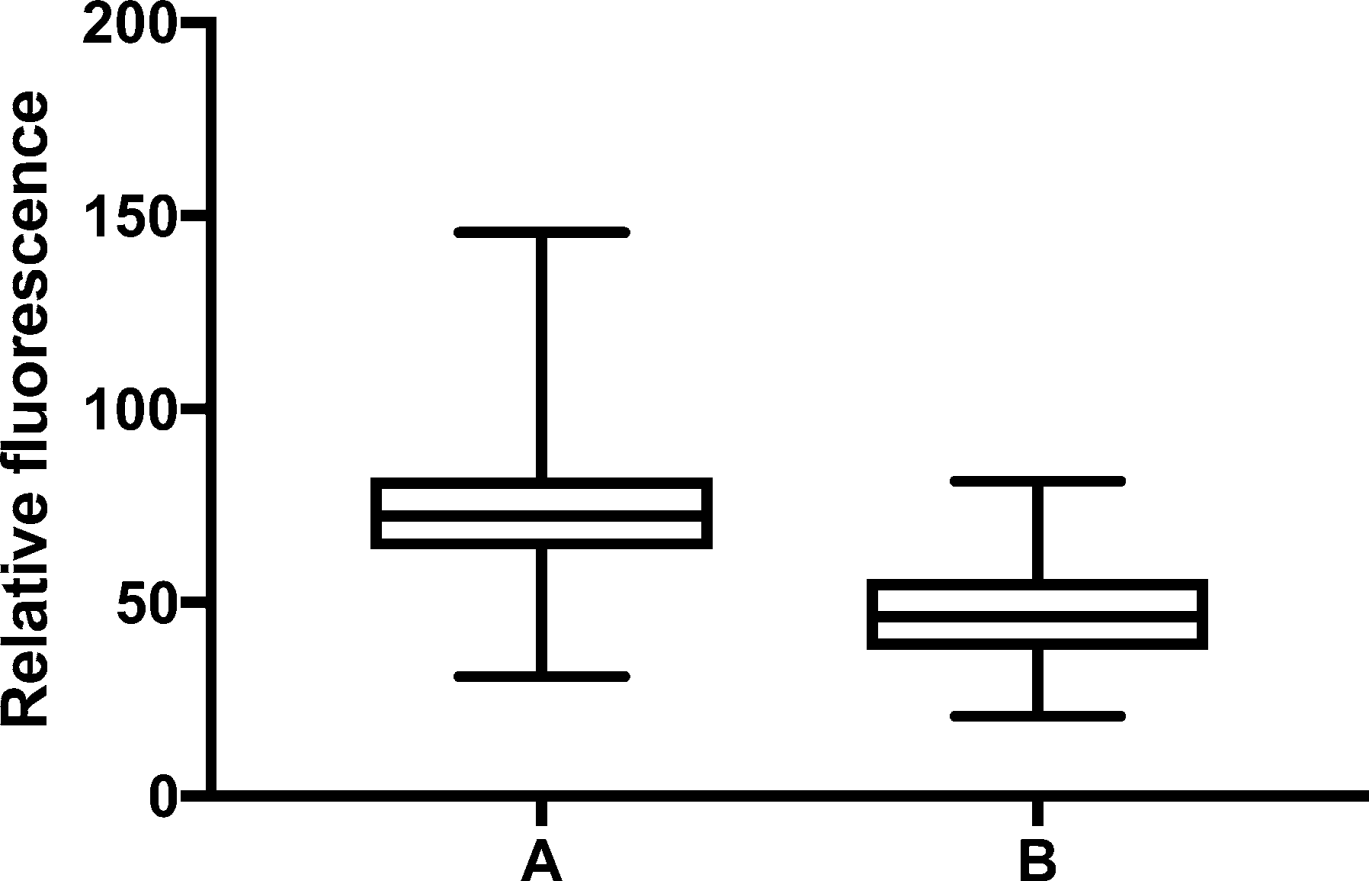
Single-cell fluorescence intensity of *E. coli* BW25113 expressing mScarlet red fluorescent protein under the control of the (**A**) *tolC* promoter and (**B**) *soxS* promoter. The bar in the box depicts the median and the bar above and below the box shows the maximum and minimum values respectively

**Fig. 4:**
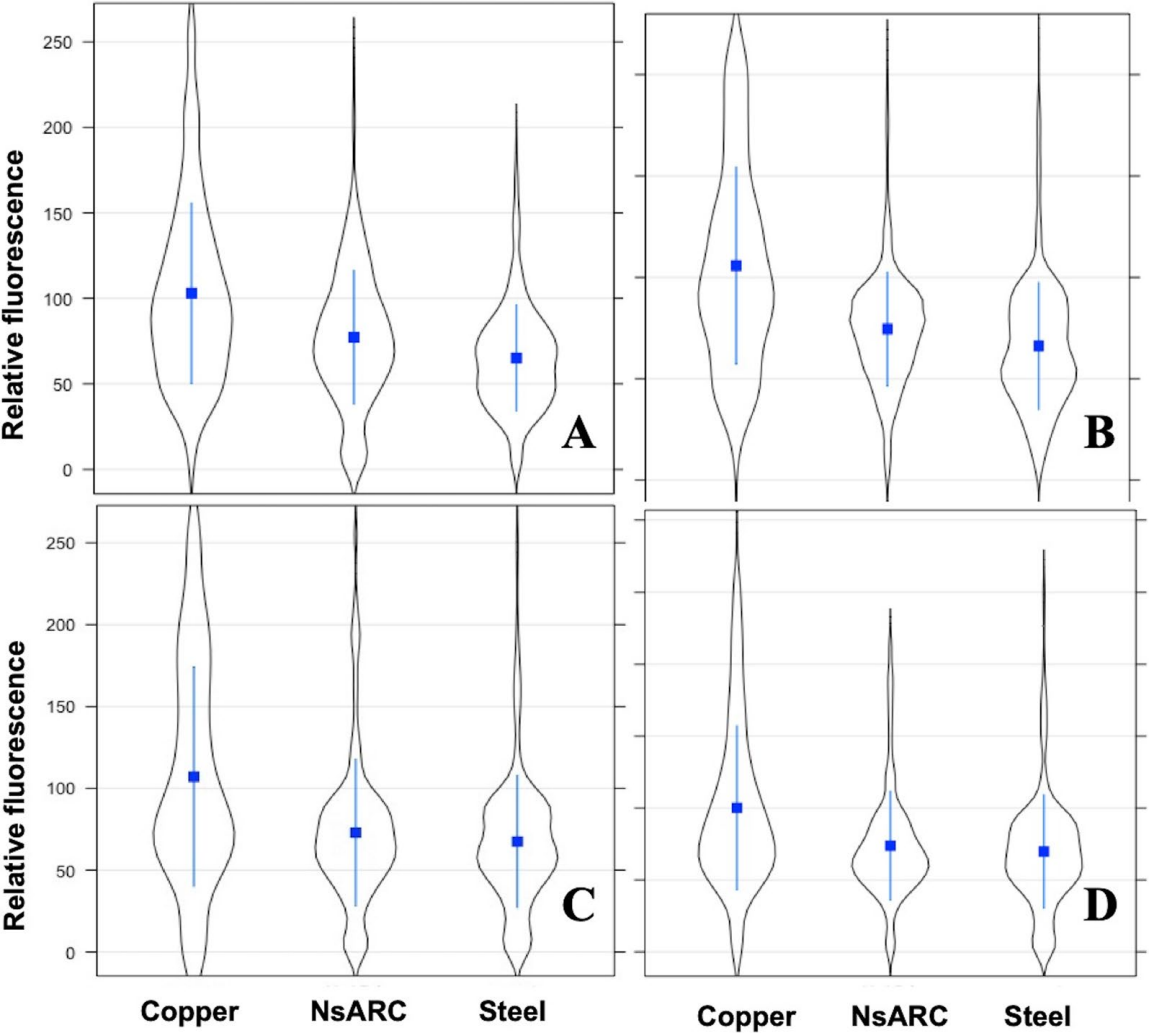
Single-cell fluorescence intensity of *E. coli* BW25113 expressing mScarlet red fluorescent protein under the control of the *tolC* promoter upon exposure to copper, NsARC and stainless steel under (**A**) UV light, (**B**) visible light, (**C**) ambient light and (**D**) dark. The violin plots show the distribution of the single-cell fluorescence within the cell population. The bar in the box depicts the median and the box shows the 25% and 75% quartiles

**Fig. 5:**
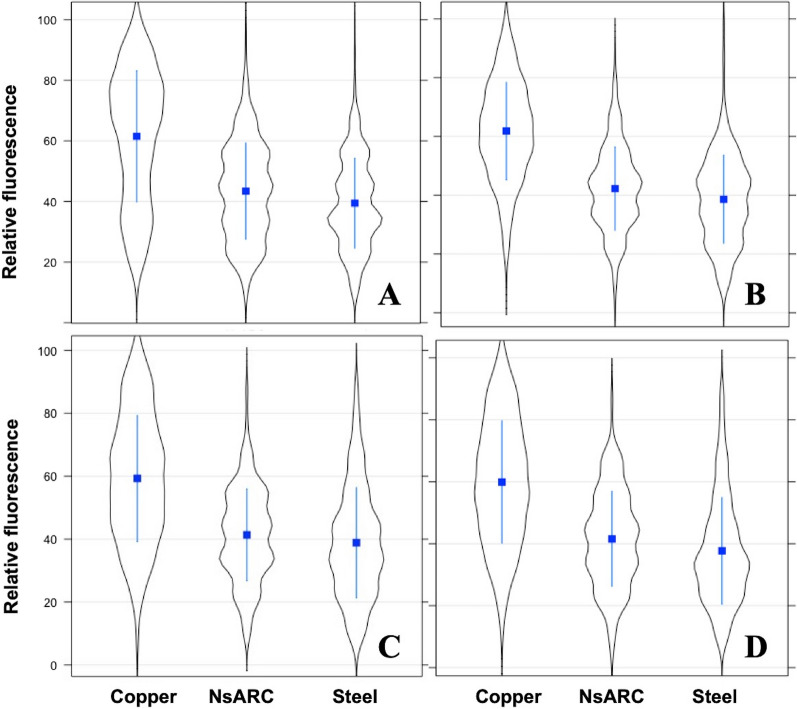
Single-cell fluorescence intensity of *E. coli* BW25113 expressing mScarlet red fluorescent protein under the control of the *soxS* promoter upon exposure to copper, NsARC and stainless steel under (**A**) UV light, (**B**) visible light, (**C**) ambient light and (**D**) dark. The violin plots show the distribution of the single-cell fluorescence within the cell population. The bar in the box depicts the median and the box shows the 25% and 75% quartiles

Overall, the *PtolC*_*−*_mScarlet reporter strain was brighter than the reporter strain expressing *PsoxS*_*−*_mScarlet (Fig. 3). There were also variations in the fluorescence of both reporters when exposed to either NsARC, stainless steel or copper regardless of light conditions (high intensity visible, UV, ambient light and no light) (*P* < 0.001) indicating that both material type and exposure conditions contribute to the variation in fluorescence intensities. Both reporter strains were brighter on copper under all exposure conditions compared to when they were on either NsARC or stainless steel (Figs. 4, 5).

The reporter strain expressing *PtolC*_*−*_mScarlet and exposed to UV light (Fig. 4A) was significantly brighter on copper than when exposed to UV and NsARC (*P* < 0.001) or UV and stainless steel (*P* < 0.001). The bacteria that were on NsARC were significantly brighter (*P* < 0.001) than the ones that were on stainless steel. Under high-intensity visible light (Fig. 4B), reporter strains that were on NsARC were significantly brighter (*P* < 0.001) than the ones on stainless steel. However, under ambient light (Fig. 4C), reporter strains on NsARC were not significantly brighter (*P* = 0.357) than on steel. In the dark (Fig. 4D) reporter strains on NsARC were not significantly brighter (*P* = 0.479) than the ones on stainless steel.

Under all light exposures *PsoxS-*mScarlet reporter strains were brighter on copper than on either NsARC of stainless steel. Combined UV light and NsARC exposures (Fig. 5A) caused greater fluorescence than combined UV light and stainless steel (*P* < 0.001). High-intensity visible light and NsARC caused greater fluorescence (*P* = 0.003) than combined UV light and stainless steel (Fig. 5B). Fluorescence for combined ambient light and NsARC exposures was also greater than (*P* = 0.010) combined ambient light and stainless steel exposures (Fig. 5C). Finally, in the dark (Fig. 5D), reporter strains were significantly brighter (*P* = 0.009) on NsARC than on stainless steel.

## Discussion

*E. coli* and *S. aureus* exposed to combinations of non-antibiotic antimicrobials and antibiotics demonstrate altered responses to antibiotics (Alderton et al. 2021). The responses can result in bacteria that survive at antibiotic concentrations above the previous minimal inhibitory concentration, have a survival or growth advantage at sub-lethal concentrations, or the opposite in that they die at lower concentrations or have a growth disadvantage at lower concentrations of antibiotic. In all cases the frequency of evolution of clinically relevant resistance increases when the bacteria are exposed to antibiotics at concentrations that alter their fitness (Alderton et al. 2021; Kurenbach et al. 2018).

It is unknown if exposures to coating materials have a similar effect on microbes. We tested this using *E. coli* and *S. aureus* that have survived exposure to NsARC. We observed that there was a significant increase in susceptibility of *E. coli* to one antibiotic, kanamycin, with no significant change in susceptibility to the other antibiotics tested. There was also no change in the susceptibility of *S. aureus* to any of the antibiotics that were tested after exposure to NsARC.

We also used fluorescent reporter strains to determine if *tolC* and *soxS* genes were transcriptionally induced by exposures to copper or NsARC. Reporters that were exposed to copper were significantly brighter than when exposed to NsARC or stainless steel. The increase in fluorescence of the reporter strains that were exposed to copper is not surprising because we have earlier reported a similar increase in fluorescence when the reporter strain expressing *PtolC*_*−*_mScarlet was cultured in media supplemented with copper (Jun et al. 2019). Copper is a known inducer of *soxS* and *tolC* transcription (Franke et al. 2003; Nishino et al. 2007), and can also induce *marR*, making *marR* an agent that acts as a copper sensor (Hao et al. 2014). The increase in cell fluorescence may be due to oxidative activity of the copper (Fenoglio et al. 2009).

Reporter strains that were exposed to NsARC and UV or high intensity light were significantly brighter than the strains that were on stainless under the same conditions. Fluorescence levels were the same for bacteria that were exposed to either NsARC or stainless steel and either ambient light or no light. The major reason for the difference in brightness in the presence or absence of light is not known, but possibly it is because light excitation of NsARC is needed in order to facilitate the production of ROS and other free radicals that can induce *soxS* and *tolC* genes (Koutsolioutsou et al. 2005; Verdier et al. 2014). Absence of light excitation affects the production of free radicals and hence these gene are not induced with no corresponding effect on the brightness of the cell.

Aside from the potential usefulness of NsARC for its antimicrobial properties, there was a need to also consider the possible potential adverse effects NsARC may have. NsARC is a biocidal material, and like other antimicrobial agents, there is a possibility that it may induce higher levels of antibiotic resistance. The fact that the reporter strains that were on NsARC were brighter than the ones on stainless steel indicated that NsARC may be able to induce genes that also have effects on antibiotic toxicities. From this study, we could not explain the mechanism by which NsARC was inducing the genes because we only tested a specific efflux pump component and a regulatory protein. We also could not quantify how much of this increase in fluorescence accounts for the increase in the expression of efflux pumps. Whole transcriptome sequencing could provide an overview of mRNA levels of most genes that might be affected by NsARC.

## Supplementary information

**Additional file 1: Fig. S1**.Response of E. coli to various concentrations of (A) Tetracycline (C)Chloramphenicol (E) Ciprofloxacin after surviving exposure to stainless steeland NsARC in the dark and (B) Tetracycline (D) Chloramphenicol (F)Ciprofloxacin after surviving exposure to stainless steel and NsARC undervisible light. **Table S1** Bonferronicontrasts (light) for E.coli survivors challenged with kanamycin. **Table S2** Bonferroni contrasts (Dark) for E. coli survivors challenged with kanamycin. **Table S3** Bonferroni contrasts for E. coli survivors challenged with tetracycline. **Table S4** Bonferroni contrasts for E. coli survivors challenged with chloramphenicol. **Table S5** Bonferronicontrasts for *E. coli*survivors challenged with ciprofloxacin. **Figure. S2** Response of S. aureus tovarious concentrations of (A) kanamycin (C) vancomycin (E) erythromycin (G)oxacillin (I)tetracycline (K) fusidic acid after surviving exposure tostainless steel and NsARC in the dark and (B) kanamycin (D) vancomycin (F)erythromycin (H) oxacillin (J) tetracycline (L) fusidic acid after survivingexposure to stainless steel and NsARC under visible light. **TableS6** Bonferroni contrasts (Dark) for *S. aureus*survivors challenged with kanamycin. **Table S7** Bonferronicontrasts (light) for *S. aureus*survivors challenged with kanamycin. **Table S8** Bonferronicontrasts for *S. aureus*survivors challenged with vancomycin. **Table S9** Bonferronicontrasts* S. aureus*survivors challenged with erythromycin. **TableS10** Bonferroni contrasts for *S. aureus*survivors challenged with oxacillin. **Table S11** Bonferronicontrasts for *S. aureus*survivors challenged with tetracycline. **TableS12** Bonferroni contrasts for *S. aureus*survivor challenged with fusidic acid. **TableS13** Tukey’s contrasts for *E. coli *expressing *PtolC-*mScarletunderuv light. **Table S14** Tukey’s contrasts for *E. coli *expressing*PtolC-*mScarlet Under high intensityvisible light. **Table S15** Tukey’s contrasts for *E. coli *expressing *PtolC-*mScarletunderAmbient light. **Table S16** Tukey’s contrasts for *E. coli *expressing *PtolC-*mScarletinthe dark. **Table S17** Tukey’s contrasts for *E. coli *expressing *PsoxS-*mScarletunderuv light. **Table S18** Tukey’s contrasts for *E. coli *expressing *PsoxS-*mScarletunderhigh intensity visible light. **Table S19** Tukey’s contrasts for *E. coli *expressing*PsoxS-*mScarlet under Ambient light. **TableS20** Tukey’s contrasts for *E. coli *expressing *PsoxS-*mScarletinthe dark. **Table S21**: The minimumconcentration (MIC) of each antibiotic in µg/ml necessary to reducethe EOP of each test organism by at least 1000-fold. NT=not tested.

## Data Availability

Authors can confirm that all relevant data are included in the article and/or its supplementary information files.
