# Supplementary material for: Copper and nanostructured anatase rutile and carbon coatings induce adaptive antibiotic resistance
Source: AMB Express. 2022 Sep 7;12:117. doi: 10.1186/s13568-022-01457-z (PMC9452618; doi:10.1186/s13568-022-01457-z)
Supplement: Supplementary file 1 — Additional file 1: Fig. S1.Response of E. coli to various concentrations of (A) Tetracycline (C)Chloramphenicol (E) Ciprofloxacin after surviving exposure to stainless steeland NsARC in the dark and (B) Tetracycline (D) Chloramphenicol (F)Ciprofloxacin after surviving exposure to stainless steel and NsARC undervisible light. Table S1 Bonferronicontrasts (light) for E.coli survivors challenged with kanamycin. Table S2 Bonferroni contrasts (Dark) for E. coli survivors challenged with kanamycin. Table S3 Bonferroni contrasts for E. coli survivors challenged with tetracycline. Table S4 Bonferroni contrasts for E. coli survivors challenged with chloramphenicol. Table S5 Bonferronicontrasts for E. colisurvivors challenged with ciprofloxacin. Figure. S2 Response of S. aureus tovarious concentrations of (A) kanamycin (C) vancomycin (E) erythromycin (G)oxacillin (I)tetracycline (K) fusidic acid after surviving exposure tostainless steel and NsARC in the dark and (B) kanamycin (D) vancomycin (F)erythromycin (H) oxacillin (J) tetracycline (L) fusidic acid after survivingexposure to stainless steel and NsARC under visible light. TableS6 Bonferroni contrasts (Dark) for S. aureussurvivors challenged with kanamycin. Table S7 Bonferronicontrasts (light) for S. aureussurvivors challenged with kanamycin. Table S8 Bonferronicontrasts for S. aureussurvivors challenged with vancomycin. Table S9 Bonferronicontrasts S. aureussurvivors challenged with erythromycin. TableS10 Bonferroni contrasts for S. aureussurvivors challenged with oxacillin. Table S11 Bonferronicontrasts for S. aureussurvivors challenged with tetracycline. TableS12 Bonferroni contrasts for S. aureussurvivor challenged with fusidic acid. TableS13 Tukey’s contrasts for E. coli expressing PtolC-mScarletunderuv light. Table S14 Tukey’s contrasts for E. coli expressingPtolC-mScarlet Under high intensityvisible light. Table S15 Tukey’s contrasts for E. coli expressing PtolC-mScarletunderAmbient light. Table S16 Tukey’s contras [file 13568_2022_1457_MOESM1_ESM.docx]

**Applied Microbiology and Biotechnology**

**Copper and Nanostructured Anatase Rutile and Carbon coatings induce adaptive antibiotic resistance**

**Alibe Wasa^1^, Jack Aitken^1^, Hyunwoo Jun^1^, Catherine Bishop^2^, Susan Krumdieck^2^, William Godsoe^3^ and Jack A. Heinemann^1^**

^1^School of Biological Sciences, University of Canterbury, New Zealand.

^2^Department of Mechanical Engineering, University of Canterbury, New Zealand.

^3^Bio-Protection Centre, Lincoln University, New Zealand.

**Corresponding email:** phone +643 369 5597 [jack.heinemann@canterbury.ac.nz](mailto:jack.heinemann@canterbury.ac.nz)

**Supplementary Material**

**Investigating changes in susceptibility to antibiotics**

*E. coli* that survived exposure to NsARC in the dark and light were challenged with different concentrations of tetracycline, chloramphenicol and ciprofloxacin. As shown in Fig. S1. Bonferroni’s post hoc test was used to compare the EOPs to determine if there is a significant difference in the EOPs, which will indicate a significant difference in the susceptibility of *E. coli* that survived exposure to NsARC and stainless steel before being challenged with the various antibiotics. When we compared the survivors that were on NsARC and stainless steel challenged with < 4 µg/ml kanamycin (Table S1 and S2), there was no significant difference in the EOPs, indicating no significant change in susceptibility. But at concentrations > 4 µg/ml (Table S1 and S2), there was a significant difference in the EOPs suggesting a significant change in susceptibility to kanamycin at the higher concentrations. No such change in EOPs was observed for the survivors that were challenged with tetracycline (Table S3), chloramphenicol (Table S4) and ciprofloxacin (Table S5), suggesting that there was no significant change in susceptibility.

**Figure. S1: Response of E. coli to various concentrations of (A) Tetracycline (C) Chloramphenicol (E) Ciprofloxacin after surviving exposure to stainless steel and NsARC in the dark and (B) Tetracycline (D) Chloramphenicol (F) Ciprofloxacin after surviving exposure to stainless steel and NsARC under visible light.**

**Table S1 Bonferroni contrasts (light) for *E. coli* survivors challenged with kanamycin**

**Linear Hypotheses:**

Estimate Std. Error t value Pr(>|t|)

1(NsARC_0 vs Steel_0) == 0 -0.32304 0.22598 -1.429 0.637

2 (NsARC_4 vs Steel_4)== 0 0.07423 0.14292 0.519 0.995

3 (NsARC_5 vs Steel_5)== 0 -1.36608 0.14292 -9.558 <1e-04 ***

4 (NsARC_6 vs Steel_6)== 0 -1.54810 0.14292 -10.832 <1e-04 ***

5 (NsARC_8 vs Steel_8)== 0 -1.00811 0.14292 -7.053 <1e-04 ***

6 (NsARC_9 vs Steel_9)== 0 0.20834 0.14292 1.458 0.617

Signif. codes: 0 ‘***’ 0.001 ‘**’ 0.01 ‘*’ 0.05 ‘.’ 0.1 ‘ ’ 1

**Table S2 Bonferroni contrasts (Dark) for *E. coli* survivors challenged with kanamycin**

**Linear Hypotheses:**

Estimate Std. Error t value Pr(>|t|)

1 (NsARC_0 vs Steel_0)== 0 -4.127e-01 1.506e-01 -2.741 0.064 .

2 (NsARC_4 vs Steel_4) == 0 -1.076e+00 9.523e-02 -11.302 <1e-04 ***

3 (NsARC_5 vs Steel_5)== 0 -1.917e+00 9.523e-02 -20.132 <1e-04 ***

4 (NsARC_6 vs Steel_6)== 0 -2.752e+00 9.523e-02 -28.894 <1e-04 ***

5 (NsARC_8 vs Steel_8)== 0 -4.441e-16 9.523e-02 0.000 1.000

6 (NsARC_9 vs Steel_9)== 0 -6.217e-15 9.523e-02 0.000 1.000

Signif. codes: 0 ‘***’ 0.001 ‘**’ 0.01 ‘*’ 0.05 ‘.’ 0.1 ‘ ’ 1

**Table S3 Bonferroni contrasts for *E. coli* survivors challenged with tetracycline**

**Linear Hypotheses:**

Estimate Std. Error t value Pr(>|t|)

1 (NsARC_0 vs Steel_0)== 0 -2.827e-01 1.602e-01 -1.765 0.412

2 (NsARC_0.5 vs Steel_0.5)== 0 -1.527e-01 1.013e-01 -1.507 0.583

3 (NsARC_0.7 vs Steel_0.7)== 0 -1.013e-01 1.013e-01 -0.999 0.893

4 (NsARC_1 vs Steel_1)== 0 -2.363e-01 1.013e-01 -2.332 0.151

5 (NsARC_1.5 vs Steel_1.5)== 0 -1.159e-01 1.013e-01 -1.144 0.820

6 (NsARC_2 vs Steel_2)== 0 -4.441e-15 1.013e-01 0.000 1.000

Signif. codes: 0 ‘***’ 0.001 ‘**’ 0.01 ‘*’ 0.05 ‘.’ 0.1 ‘ ’ 1

**Table S4 Bonferroni contrasts for *E. coli* survivors challenged with chloramphenicol**

**Linear Hypotheses:**

Estimate Std. Error t value Pr(>|t|)

1 (NsARC_0 vs Steel_0)== 0 -3.859e-01 2.725e-01 -1.416 0.646

2 (NsARC_5 vs Steel_5)== 0 8.509e-02 1.723e-01 0.494 0.996

3 (NsARC_6 vs Steel_6)== 0 -3.750e-01 1.723e-01 -2.176 0.205

4 (NsARC_7 vs Steel_7)== 0 -3.427e-01 1.723e-01 -1.988 0.287

5 (NsARC_8 vs Steel_8)== 0 3.000e-01 1.723e-01 1.741 0.427

6 (NsARC_9 vs Steel_9)== 0 -4.441e-15 1.723e-01 0.000 1.000

Signif. codes: 0 ‘***’ 0.001 ‘**’ 0.01 ‘*’ 0.05 ‘.’ 0.1 ‘ ’ 1

**Table S5 Bonferroni contrasts for *E. coli* survivors challenged with ciprofloxacin**

**Linear Hypotheses:**

Estimate Std. Error t value Pr(>|t|)

1 (NsARC_0 vs Steel_0) == 0 8.606e-02 1.712e-01 0.503 0.996

2 (NsARC_0.1 vs Steel_0.1)== 0 1.491e-01 1.083e-01 1.377 0.673

3 (NsARC_0.2 vs Steel_0.2)== 0 -1.073e-02 1.083e-01 -0.099 1.000

4 (NsARC_0.3 vs Steel_0.3)== 0 -8.882e-16 1.083e-01 0.000 1.000

5 (NsARC_0.4 vs Steel_0.4)== 0 1.776e-15 1.083e-01 0.000 1.000

6 (NsARC_0.5 vs Steel_0.5)== 0 -6.217e-15 1.083e-01 0.000 1.000

Signif. codes: 0 ‘***’ 0.001 ‘**’ 0.01 ‘*’ 0.05 ‘.’ 0.1 ‘ ’ 1

*S. aureus* that survived exposure to NsARC in the dark and light were also challenged with different concentrations of kanamycin, vancomycin, erythromycin, oxacillin, tetracycline and fusidic acid. As shown in Fig. S2. We also conducted a Bonferroni’s post hoc test to compare the EOPs to determine if there is a significant difference in the EOPs, which will indicate a significant difference in the susceptibility of *S. aureus* that survived exposure to NsARC and stainless steel before being challenged with the various antibiotics. We observed that there was no change in EOPs for the survivors that were challenged with kanamycin (Table S6 and S7), vancomycin (Table S8), erythromycin (Table S9), oxacillin (Table S10), tetracycline (Table S11) and fusidic acid (Table S12), suggesting that there was no significant change in susceptibility.

**Figure. S2: Response of S. aureus to various concentrations of (A) kanamycin (C) vancomycin (E) erythromycin (G) oxacillin (I)tetracycline (K) fusidic acid after surviving exposure to stainless steel and NsARC in the dark and (B) kanamycin (D) vancomycin (F) erythromycin (H) oxacillin (J) tetracycline (L) fusidic acid after surviving exposure to stainless steel and NsARC under visible light**

**Table S6 Bonferroni contrasts (Dark) for *S. aureus* survivors challenged with kanamycin**

**Linear Hypotheses:**

Estimate Std. Error t value Pr(>|t|)

1 (NsARC_0 vs Steel_0)== 0 2.443e-01 1.461e-01 1.672 0.471

2 (NsARC_0.9 vs Steel_0.9)== 0 9.527e-03 9.241e-02 0.103 1.000

3 (NsARC_1 vs Steel_1)== 0 -8.775e-02 9.241e-02 -0.950 0.913

4 (NsARC_2 vs Steel_2)== 0 -1.003e-01 9.241e-02 -1.086 0.852

5 (NsARC_3 vs Steel_3)== 0 1.776e-15 9.241e-02 0.000 1.000

6 (NsARC_4 vs Steel_4)== 0 -7.105e-15 9.241e-02 0.000 1.000

Signif. codes: 0 ‘***’ 0.001 ‘**’ 0.01 ‘*’ 0.05 ‘.’ 0.1 ‘ ’ 1

**Table S7 Bonferroni contrasts (light) for *S. aureus* survivors challenged with kanamycin**

**Linear Hypotheses:**

Estimate Std. Error t value Pr(>|t|)

1 (NsARC_0 vs Steel_0)== 0 -2.318e-01 1.193e-01 -1.943 0.3099

2 (NsARC_0.9 vs Steel_0.9)== 0 -3.678e-02 7.545e-02 -0.487 0.9967

3 (NsARC_1 vs Steel_1)== 0 -2.330e-01 7.545e-02 -3.088 0.0289 *

4 (NsARC_2 vs Steel_2)== 0 -2.665e-15 7.545e-02 0.000 1.0000

5 (NsARC_3 vs Steel_3)== 0 0.000e+00 7.545e-02 0.000 1.0000

6 (NsARC_4 vs Steel_4)== 0 -3.553e-15 7.545e-02 0.000 1.0000

Signif. codes: 0 ‘***’ 0.001 ‘**’ 0.01 ‘*’ 0.05 ‘.’ 0.1 ‘ ’ 1

**Table S8 Bonferroni contrasts for *S. aureus* survivors challenged with vancomycin**

**Linear Hypotheses:**

Estimate Std. Error t value Pr(>|t|)

1 (NsARC_0 vs Steel_0)== 0 -7.521e-03 1.239e-01 -0.061 1.000

2 (NsARC_0.5 vs Steel_0.5)== 0 -3.934e-02 7.835e-02 -0.502 0.996

3 (NsARC_0.8 vs Steel_0.8)== 0 -8.724e-02 7.835e-02 -1.113 0.837

4 (NsARC_1 vs Steel_1)== 0 -8.083e-01 7.835e-02 -10.316 <1e-04 ***

5 (NsARC_1.5 vs Steel_1.5)== 0 8.882e-16 7.835e-02 0.000 1.000

6 (NsARC_2 vs Steel_2)== 0 -4.441e-15 7.835e-02 0.000 1.000

Signif. codes: 0 ‘***’ 0.001 ‘**’ 0.01 ‘*’ 0.05 ‘.’ 0.1 ‘ ’ 1

**Table S9 Bonferroni contrasts *S. aureus* survivors challenged with erythromycin**

**Linear Hypotheses:**

Estimate Std. Error t value Pr(>|t|)

1 (NsARC_0 vs Steel_0)== 0 -9.706e-03 2.329e-01 -0.042 1.000000

2 (NsARC_1 vs Steel_1)== 0 -7.292e-01 1.473e-01 -4.950 0.000262 ***

3 (NsARC_2 vs Steel_2)== 0 -1.804e-01 1.473e-01 -1.225 0.772686

4 (NsARC_4 vs Steel_4)== 0 -2.817e-01 1.473e-01 -1.912 0.326368

5 (NsARC_5 vs Steel_5)== 0 -1.451e-01 1.473e-01 -0.985 0.898721

6 (NsARC_6 vs Steel_6)== 0 -4.441e-15 1.473e-01 0.000 1.000000

Signif. codes: 0 ‘***’ 0.001 ‘**’ 0.01 ‘*’ 0.05 ‘.’ 0.1 ‘ ’ 1

**Table S10 Bonferroni contrasts for *S. aureus* survivors challenged with oxacillin**

**Linear Hypotheses:**

Estimate Std. Error t value Pr(>|t|)

1 (NsARC_0 vs Steel_0)== 0 -1.232e-01 1.179e-01 -1.045 0.8719

2 (NsARC_0.07 vs Steel_0.07)== 0 -6.151e-02 7.455e-02 -0.825 0.9530

3 (NsARC_0.08 vs Steel_0.08)== 0 -3.923e-03 7.455e-02 -0.053 1.0000

4 (NsARC_0.09 vs Steel_0.09)== 0 -2.027e-08 7.455e-02 0.000 1.0000

5 (NsARC_0.1 vs Steel_0.1)== 0 -2.123e-01 7.455e-02 -2.847 0.0503 .

6 (NsARC_0.2 vs Steel_0.2)== 0 -5.329e-15 7.455e-02 0.000 1.0000

Signif. codes: 0 ‘***’ 0.001 ‘**’ 0.01 ‘*’ 0.05 ‘.’ 0.1 ‘ ’ 1

**Table S11 Bonferroni contrasts for *S. aureus* survivors challenged with tetracycline**

**Linear Hypotheses:**

Estimate Std. Error t value Pr(>|t|)

1 (NsARC_0 vs Steel_0)== 0 -3.760e-03 9.339e-02 -0.040 1.0000

2 (NsARC_0.09 vs Steel_0.09)== 0 -1.757e-02 5.907e-02 -0.297 0.9998

3 (NsARC_0.1 vs Steel_0.1)== 0 6.588e-02 5.907e-02 1.115 0.8362

4 (NsARC_0.2 vs Steel_0.2)== 0 1.624e-01 5.907e-02 2.750 0.0527 *.

5 (NsARC_0.3 vs Steel_0.3)== 0 2.469e-02 5.907e-02 0.418 0.9986

6 (NsARC_0.4 vs Steel_0.4)== 0 -7.105e-15 5.907e-02 0.000 1.0000

Signif. codes: 0 ‘***’ 0.001 ‘**’ 0.01 ‘*’ 0.05 ‘.’ 0.1 ‘ ’ 1

**Table S12 Bonferroni contrasts for *S. aureus* survivor challenged with fusidic acid**

**Linear Hypotheses:**

Estimate Std. Error t value Pr(>|t|)

1 (NsARC_0 vs Steel_0)== 0 -3.213e-01 1.087e-01 - 2.957 0.0393 *

2 (NsARC_0.09 vs Steel_0.09)== 0 1.089e-01 6.872e-02 1.584 0.5298

3 (NsARC_0.1 vs Steel_0.1)== 0 5.544e-02 6.872e-02 0.807 0.9576

4 (NsARC_0.2 vs Steel_0.2)== 0 -4.441e-16 6.872e-02 0.000 1.0000

5 (NsARC_0.3 vs Steel_0.3)== 0 4.441e-16 6.872e-02 0.000 1.0000

6 (NsARC_0.4 vs Steel_04.)== 0 -5.329e-15 6.872e-02 0.000 1.0000

Signif. codes: 0 ‘***’ 0.001 ‘**’ 0.01 ‘*’ 0.05 ‘.’ 0.1 ‘ ’ 1

**Investigating if *tol*C and *sox*S can be induced by NsARC**

A Tukey’s post hoc test was used to compare the relative fluorescence of *E. coli* expressing *PtolC-*mScarlet and *PsoxS-*mScarlet that was exposed to copper, NsARC and stainless steel under uv light, High intensity visible light, ambient light and in the dark. The differences between individual treatment combinations, such as NsARC vs Positive control (copper), Negative control (stainless steel) vs Positive control (copper) and NsARC vs Negative control (stainless steel) was what we were interested in. These differences were calculated using contrasts and the results as follows (Figure S13-S20).

**Table S13 Tukey’s contrasts for *E. coli* expressing *PtolC-*mScarlet under uv light**

**Linear Hypotheses**:

Estimate Std. Error t value Pr(>|t|)

NsARC - copper == 0 -25.839 3.227 -8.007 < 1e-04 ***

steel – copper == 0 -37.902 3.303 -11.477 < 1e-04 ***

steel - NsARC == 0 -12.063 3.177 -3.797 0.000458 ***

Signif. codes: 0 ‘***’ 0.001 ‘**’ 0.01 ‘*’ 0.05 ‘.’ 0.1 ‘ ’ 1

**Table S14 Tukey’s contrasts for *E. coli* expressing *PtolC-*mScarlet Under high intensity visible light**

**Linear Hypotheses:**

Estimate Std. Error t value Pr(>|t|)

NsARC - copper == 0 -31.269 2.088 -14.975 < 1e-09 ***

steel - copper == 0 -39.597 2.065 -19.175 < 1e-09 ***

steel - NsARC == 0 -8.328 1.331 -6.257 1.36e-09 ***

Signif. codes: 0 ‘***’ 0.001 ‘**’ 0.01 ‘*’ 0.05 ‘.’ 0.1 ‘ ’ 1

**Table S15 Tukey’s contrasts for *E. coli* expressing *PtolC-*mScarlet under Ambient light**

**Linear Hypotheses:**

Estimate Std. Error t value Pr(>|t|)

NsARC – copper == 0 -34.162 3.980 -8.584 <1e-04 ***

steel - copper == 0 -39.613 3.980 -9.954 <1e-04 ***

steel - NsARC == 0 -5.451 3.980 -1.370 0.357

Signif. codes: 0 ‘***’ 0.001 ‘**’ 0.01 ‘*’ 0.05 ‘.’ 0.1 ‘ ’ 1

**Table S16 Tukey’s contrasts for *E. coli* expressing *PtolC-*mScarlet in the dark**

**Linear Hypotheses**:

Estimate Std. Error t value Pr(>|t|)

NsARC - copper == 0 -26.378 3.487 -7.564 <1e-05 ***

steel - copper == 0 -30.411 3.487 -8.721 <1e-05 ***

steel - NsARC == 0 -4.034 3.487 -1.157 0.479

Signif. codes: 0 ‘***’ 0.001 ‘**’ 0.01 ‘*’ 0.05 ‘.’ 0.1 ‘ ’ 1

**Table S17 Tukey’s contrasts for *E. coli* expressing *PsoxS-*mScarlet under uv light**

**Linear Hypotheses**:

Estimate Std. Error t value Pr(>|t|)

NsARC - copper == 0 -18.0874 0.9707 -18.634 < 1e-04 ***

steel - copper == 0 -22.0462 0.9707 -22.713 < 1e-04 ***

steel - NsARC == 0 -3.9588 0.9707 -4.078 0.000149 ***

Signif. codes: 0 ‘***’ 0.001 ‘**’ 0.01 ‘*’ 0.05 ‘.’ 0.1 ‘ ’ 1

**Table S18 Tukey’s contrasts for *E. coli* expressing *PsoxS-*mScarlet under high intensity visible light**

**Linear Hypotheses**:

Estimate Std. Error t value Pr(>|t|)

NsARC - copper == 0 -19.617 1.098 -17.859 < 0.001 ***

steel - copper == 0 -23.228 1.098 -21.146 < 0.001 ***

steel - NsARC == 0 -3.611 1.098 -3.288 0.00305 **

Signif. codes: 0 ‘***’ 0.001 ‘**’ 0.01 ‘*’ 0.05 ‘.’ 0.1 ‘ ’ 1

**Table S19 Tukey’s contrasts for *E. coli* expressing *PsoxS-*mScarlet under Ambient light**

**Linear Hypotheses:**

Estimate Std. Error t value Pr(>|t|)

NsARC - copper == 0 -17.945 1.231 -14.58 <1e-04 ***

steel - copper == 0 -20.444 1.231 -16.61 <1e-04 ***

steel - NsARC == 0 -2.499 1.231 -2.03 0.0105 **

Signif. codes: 0 ‘***’ 0.001 ‘**’ 0.01 ‘*’ 0.05 ‘.’ 0.1 ‘ ’ 1

**Table S20 Tukey’s contrasts for *E. coli* expressing *PsoxS-*mScarlet in the dark**

**Linear Hypotheses**:

Estimate Std. Error t value Pr(>|t|)

NsARC - copper == 0 -18.358 1.302 -14.096 < 0.001 ***

steel - copper == 0 -22.178 1.302 -17.029 < 0.001 ***

steel - NsARC == 0 -3.820 1.302 -2.933 0.00957 **

Signif. codes: 0 ‘***’ 0.001 ‘**’ 0.01 ‘*’ 0.05 ‘.’ 0.1 ‘ ’ 1

**Table S21: The minimum concentration (MIC) of each antibiotic in µg/ml necessary to reduce the EOP of each test organism by at least 1000-fold. NT=not tested**

| Antibiotics | *E. coli* | *S. aureus* |
| --- | --- | --- |
| kanamycin | 6 | 1 |
| tetracycline | 1.0 | 0.1 |
| chloramphenicol | 4 | NT |
| ciprofloxacin | 0.01 | NT |
| vancomycin | NT | 0.5 |
| erythromycin | NT | 2 |
| oxacillin | NT | 0.08 |
| Fusidic acid | NT | 0.1 |
